# Intensive Local Radiotherapy Is Associated With Better Local Control and Prolonged Survival in Bone-Metastatic Nasopharyngeal Carcinoma Patients

**DOI:** 10.3389/fonc.2020.00378

**Published:** 2020-03-20

**Authors:** Xiao-Yun Li, Guo-Dong Jia, Xue-Song Sun, Shan-Shan Guo, Li-Ting Liu, Sai-Lan Liu, Jin-Jie Yan, Dong-Hua Luo, Rui Sun, Ling Guo, Hao-Yuan Mo, Lin-Quan Tang, Qiu-Yan Chen, Hai-Qiang Mai

**Affiliations:** ^1^Sun Yat-sen University Cancer Center, State Key Laboratory of Oncology in South China, Collaborative Innovation Center for Cancer Medicine, Guangdong Key Laboratory of Nasopharyngeal Carcinoma Diagnosis and Therapy, Guangzhou, China; ^2^Department of Nasopharyngeal Carcinoma, Sun Yat-sen University Cancer Center, Guangzhou, China

**Keywords:** nasopharyngeal cancer, radiotherapy, bone metastases, chemotherapy, palliaitve care

## Abstract

**Objective:** To compare the survival outcomes brought by different radiation dose schedules to bone lesions and different chemotherapy regimens in bone metastatic nasopharyngeal carcinoma (NPC).

**Background:** The current treatment strategy for bone metastatic NPC patients was empirically given and poorly studied before. It is of necessity to optimize the treatment for bone metastasis to enhance the therapeutic effect and increase the proportion of long-term survived patients.

**Methods:** Three hundred patients who received chemoradiotherapy from 2002 to 2018 were involved in the study. Demographics, laboratory results, and detailed treatment plans were recorded. Radiotherapy plans were classified into three categories based on the intensity, and the survival analysis was performed using log-rank test. Multivariable analysis was made by the Cox proportional regression model.

**Results:** Patients who received 60–75 Gy/30–35 fractions of radiation to the metastatic bones had significantly longer bone relapse-free survival (BRFS) (HR, 0.53, 95% CI, 0.37–0.78, *P* = 0.003), overall survival (OS) (HR, 0.63, 95% CI, 0.46–0.84, *P* = 0.007), and progression-free survival (PFS) (HR, 0.80, 95% CI, 0.67–0.95, *P* = 0.041). The administration of paclitaxel, cisplatin and 5-fluorouracil regimen was also associated with better BRFS (HR, 0.27, 95% CI, 0.10–0.75, *P* = 0.007), PFS (HR, 0.60, 95% CI, 0.42–0.87, *P* = 0.007), and OS with borderline significance (HR, 0.54, 95% CI, 0.29–1.03, *P* = 0.058). In multivariable analysis, the post-treatment EBV DNA level and radical radiation dose were proved as independent prognostic factors for both BRFS and OS.

**Conclusions:** Radiotherapy to metastatic bones with palliative dose prescription should not be considered in bone metastatic NPC patients. TPF chemotherapy regimen might help to improve the survivals in NPC patients but failed to be an independent protective factor.

## Introduction

Nasopharyngeal carcinoma (NPC), which stems from the epithelium of nasopharynx, is a special subtype of head and neck cancers. Characterized by the poor differentiated nature, NPC is highly sensitive to chemoradiotherapy and excellent locoregional control rates can often be achieved in non-metastatic NPC. Nonetheless, distant metastasis is the major threat and cause of death faced by all NPC patients. It's reported that nearly 10% newly-diagnosed patients presented with synchronous distant metastasis (1, 2) and approximately 20–30% patients developed metastasis after primary treatment (3–5). Bone metastasis (BM), especially axial bone, is the mostly frequently invaded organ with a proportion of over 50% (6–8) among all metastatic sites. Previous studies have shown diverse survival outcomes in this population with the median overall survival (OS) ranging from 20.3 to 36.9 months (9–13). Meanwhile, there are evidences favoring the long-term survival in some subgroups such as those with oligometastasis (11, 14), low level of pretreatment alkaline phosphatase (ALP) and Epstein-barr virus (EBV) DNA (12). However, the current treatment strategy for metastatic patients is mainly based on palliative chemotherapy, let alone the treating principle for BM, which is poorly understood. Therefore, it is of necessity to optimize the treatment of BM with different strategies considered to enhance effectiveness and increase the proportion of long-term survived patients.

Radiotherapy (RT) to metastatic bones is widely applied to relieve pain, prevent skeleton-related events (SRE) and improve quality of life among various cancer types. Accumulated evidences have pointed out the survival benefit brought by local radiotherapy to BM plus chemotherapy in NPC patients. In Liang et al.'s study (15), a significantly higher 3 year OS was found in the group receiving local treatment to metastatic sites (48.8 vs. 33.8%, *P* = 0.001), and similar results were also observed in Shen et al.'s (10) and He et al.'s study (12). Although several studies suggested the potential value of local radiotherapy to BM, no general consensus exists concerning the best candidates and the appropriate radiotherapy regimens (16). From single fraction, hypofracionation to normofractionation, radiotherapy regimens were empirically given without the underpinning of evidence, thus the optimal RT dose fractionation schedule for metastatic bones in NPC should be addressed. Similar to the situation of local therapy, the appropriate chemotherapy regimen among all platinum-based regimens for bone metastatic patients was little studied and also worth exploring.

Herein, we investigated the real-world therapeutic strategy for bone metastatic NPC patients who received chemoradiotherapy, and a retrospective cohort study was performed with an attempt to find out the optimal chemoradiation plan and yield insight into future studies to establish specific guidelines.

## Methods

### Patients

Patients treated in Sun Yat-sen University Cancer Center from January 2002 to December 2018 were consecutively evaluated for their eligibility. The diagnosis of BM was determined by at least one of the following examinations including computed tomography (CT) with contrast, magnetic resonance imaging (MRI) with contrast, positron emission tomography-computed tomography (PET/CT) and histologically proven metastatic lesion. The inclusion criteria were: (1) Patients were previously or concurrently diagnosed as NPC with pathological evidence. (2) Patients who had secondary BM received radical radiotherapy to the nasopharynx as an initial treatment. (3) Radiotherapy to the BM was performed. (4) Karnofsky performance status (KPS) ≥70. Patients were excluded if any of the following condition was met: (1) Radiotherapy was stopped halfway for any reason. (2) Coexistence of a second malignancy. (3) Incomplete medical records. (4) Patients who showed no evidence of progression were lost to follow up within 3 months after the BM-directed treatment. This study was approved by the Sun Yat-sen University Cancer Center Clinical Research Ethics Committee.

### Treatment

All patients received multi-modality treatment including radiotherapy and chemotherapy. Chemotherapy was administered every 3 weeks for at least 4–6 cycles before the radiotherapy. Chemotherapy regimens included TP, paclitaxel plus cisplatin; PF, cisplatin plus 5-fluorouracil; GP, gemcitabine plus cisplatin; and TPF, paclitaxel plus cisplatin and 5-fluorouracil. Carboplatin and nedaplatin were also applicated as substitutes for cisplatin. If several regimens were applied, the regimen with which patients achieved major response was recorded. The adopted radiotherapy techniques for BM ranged from 2-dimentional (2D-RT) or 3-dimentional radiotherapy (3D-RT), intensity modulated radiotherapy (IMRT), volumetric modulated arc therapy (VMAT) to tomotherapy (TOMO). The dose-fractionation patterns were heterogeneous among patients from single fractionation schedule to radical dose regimen. In addition, all initially diagnosed patients received radiotherapy to the nasopharynx and neck. The prescribed dose to the gross tumor volume was 66–70 Gy and 60 Gy to the clinical target volume. The zoledronic acid was given to some patients with a dosage of 4 mg every 3–4 weeks. Epidermal growth factor receptor (EGFR) inhibitors such as cetuximab and nimotuzumab, immune checkpoint inhibitors including antibodies of CTLA-4, programmed cell death receptor (PD-1) and its ligand (PD-L1) and anti-angiogenic agents like endostar and apatinib were also considered as supplement to promote the therapeutic effect.

### Data Collection

Demographics, laboratory results and detailed treatment plans of all patients were recorded. If multiple BM relapses occurred, the information collected and evaluation was subjected to the first episode. The pre-treatment levels of ALP, lactic dehydrogenase (LDH), and EBV DNA were measured at the time when the BM was detected. The plasma EBV DNA test was not performed in patients treated before 2007. The osteolytic lesion was defined when the BM appeared as disrupted trabecular structure or area of faint density, while the osteogenic lesion was distinguished by sclerotic change or enhanced density. The follow-up information of all patients was collected. The primary endpoint was *in-situ* bone relapse-free survival (BRFS), which referred to the interval from the diagnosis of BM to the *in-situ* relapse of the radiated bones. The second endpoints measured overall survival (OS) and progression-free survival (PFS), which, respectively referred to the interval from the diagnosis of BM to death caused by any reason or any tumor progression. Patients were censored at the date of last follow-up.

### Statistical Analysis

Continuous variables were dichotomized to categorical variables before analysis. The cut-off value of LDH was categorized using the upper normal limit (250 U/L), and the cut-off values of ALP and EBV DNA were, respectively determined as 110 U/L and 4,000 copies/ml based on previous literatures (2, 12, 17). The Chi-square test was adopted to calculate the correlations between variables. Survival curves were plotted with the Kaplan-meier method, and the survival outcomes were compared by the log-rank test. The univariable and multivariable analysis were conducted using the Cox proportional hazard model. All statistical analyses were carried out with the use of SPSS, version 26.0 (SPSS Inc., Chicago, IL, USA). A 2-tailed *P*-value of <0.05 was considered statistically significant.

## Results

### Patient Characteristics

**Figure 1:**
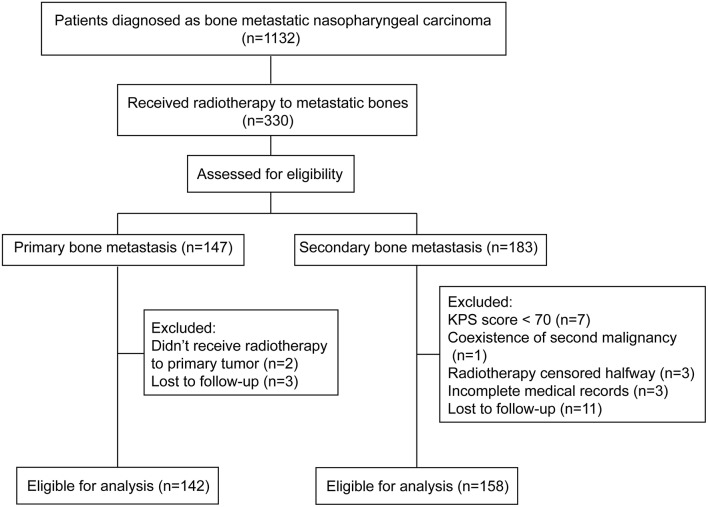
Flow diagram of study selection process. Inclusion and exclusion criteria.

Clinical data of all patients who were diagnosed with bone metastatic nasopharyngeal carcinoma in Sun Yat-sen University Cancer Center from January 2002 to December 2018 were evaluated. After the inclusion and exclusion procedure, 300 patients were eligible for analysis (Figure 1). The baseline characteristics of all patients were listed in Table 1. In brief, 142 (47.3%) patients primarily presented with bone metastasis and 158 (52.7%) were found after radical treatment. Eighty one (27.0%) patients were accompanied by concomitant metastasis of other organs. In patients with bone-only metastasis, 73.1% of them had ≤3 metastatic bones involved. The pre-treatment plasma EBV DNA level was tested in 242 patients. Two hundred and thirteen (69.4%) patients showed an EBV DNA level above 4,000 copies/ml with the median level of 10,375 copies/ml. The median follow-up time was 23.5 months (IQR, 14.7–38.5 months). The 3 year BRFS, OS and PFS rates were 84.0, 80.7, and 42.0%, respectively. No one died from radiation or chemotherapy related adverse effects. One hundred and eighty eight (62.7%) patients had disease progression and 74 patients died during the follow-up. Among patients who developed progression, 48 (25.5%) patients had bone relapse *in-situ* with or without invasions to other bones or organs.

**Table 1:** Baseline characteristics of patients.

**Variables**	**Primary bone- metastatic cohort, No (%) (*n* = 142)**	**Secondary bone- metastatic cohort, No (%) (*n* = 158)**	***P***
Follow-up, median (range)	25.2 (3.9–106.7)	22.3 (4.2–134.3)	
Gender			0.30
Male	117 (82.3)	137 (86.7)	
Female	25 (17.6)	21 (13.3)	
Age (years)			0.86
≤45	68 (47.9)	74 (46.8)	
>45	74 (52.1)	84 (53.2)	
Number of BM lesions			0.38
≤3	95 (66.9)	98 (62.0)	
>3	47 (33.1)	60 (38.0)	
Imaging feature of BM destruction			0.99
Osteogenic	79 (55.6)	88 (55.7)	
Osteolytic	63 (44.4)	70 (44.3)	
Concomitant metastasis in other organs			0.49
Yes	41 (28.9)	40 (25.3)	
No	101 (71.1)	118 (74.7)	
Pre-RT ALP level (U/L)			0.97
≤110	121 (87.1)	126 (86.9)	
>110	18 (12.9)	19 (13.1)	
Pre-RT LDH level (U/L)			0.23
≤250	112 (80.0)	123 (85.4)	
>250	28 (20.0)	21 (14.6)	
Pre-RT EBV DNA level (copies/ml)			0.08
≤4,000	43 (33.6)	51 (44.7)	
>4,000	85 (66.4)	63 (55.3)	
Dose prescription of RT			< 0.001
60–75 Gy/30–35f	61 (43.0)	34 (21.5)	
36–54 Gy/12–27f	39 (27.5)	50 (31.6)	
12–30 Gy/3–10f	39 (27.5)	65 (41.1)	
Others	3 (2.1)	9 (5.7)	
Chemotherapy regimen			< 0.001
TP	41 (28.9)	37 (23.4)	
PF	32 (22.5)	43 (27.2)	
GP	11 (7.7)	33 (20.9)	
TPF	51 (35.9)	17 (10.8)	
Others	7 (4.9)	28 (17.7)	
Other systemic therapies^*^			0.99
None	101 (71.1)	111 (70.3)	
EGFR targeted therapy	28 (19.7)	31 (19.6)	
VEGF targeted therapy	2 (1.4)	3 (1.9)	
Immunotherapy	11 (7.7)	13 (8.2)	
Administration of ZA			0.26
Yes	96 (67.6)	97 (61.4)	
No	46 (32.4)	61 (38.6)	

*BM, bone metastasis; RT, radiotherapy; ALP, alkaline phosphatase; LDH, lactic dehydrogenase; EBV, Epstein-barr virus; TP, paclitaxel plus cisplatin; PF, cisplatin plus 5-fluorouracil; GP, gemcitabine plus cisplatin; TPF, paclitaxel plus cisplatin and 5-fluorouracil; EGFR, epidermal growth factor receptor; VEGF, vascular endothelial growth factor; ZA, zoledronic acid*.

*All statistical tests were two-sided*.

* *P-value was calculated with the Fisher's exact test*.

### Radiotherapy

**Figure 2:**
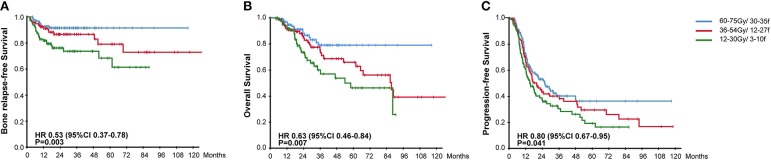
Kaplan-Meier Curves for **(A)** Bone Relapse Free Survival, **(B)** Overall Survival, **(C)** Progression Free Survival, among groups receiving different radiotherapy plans.

All patients accepted multimodality systemic treatment and radiotherapy was prescribed to the BM. The efficacy of different radiotherapy plans and chemotherapy plans were compared in Table 2. Radiotherapy was given to all metastatic bones in 208 (69.3%) patients while others had radiotherapy for palliative pain alleviation. Radiotherapy plans were classified into three categories based on the intensity. 60–75 Gy using 30–35 fractions was considered radical dose prescription. Meanwhile, 36–54 Gy with 12–27 fractions and 12–30 Gy with 3–10 fractions were, respectively categorized into moderate and palliative dose prescription. There were accordingly 95 (31.7%), 89 (29.7%), and 104 (34.7%) patients receiving the above-mentioned plans. The hypofractionated and single fractionated regimens were seldomly used. As the radiotherapy plans for 12 patients failed to be classified, those plans were listed separately in the Supplementary Table 1. The survival results were compared among the three groups, patients given radical radiation dose were associated with significantly higher BRFS (91.6 vs. 84.3 vs. 75.0%, HR, 0.53, 95% CI, 0.37–0.78, *P* = 0.003), OS (84.2 vs. 69.7 vs. 70.2%, HR, 0.63, 95% CI, 0.46–0.84, *P* = 0.007), and PFS (43.2 vs. 33.7% vs. 31.7, HR, 0.80, 95% CI, 0.67–0.95, *P* = 0.041) (Figure 2). In addition, a strong correlation existed between the option of radiotherapy plans and the incidence of post-treatment EBV DNA levels dropping to zero (HR, 0.58, 95% CI, 0.40–0.83, *P* = 0.004). Concerning the effect of fractionated dose on survival, it was found that a fractionated dose of ≤ 2 Gy helped to extend the BRFS (88.5 vs. 81.3%, HR, 0.49, 95% CI, 0.26–0.94, *P* = 0.026), but brought no benefit to the OS and PFS.

**Table 2:** Summary of multivariable analyses of prognostic factors.

	**HR**	**95% CI for HR**	***P*-value**
**OVERALL SURVIVAL**			
**Radiotherapy**			
Moderate vs. radical RT	1.69	0.89–3.20	0.108
Palliative vs. radical RT	2.60	1.40–4.82	0.003
**Chemotherapy**			
TP vs. TPF	1.42	0.67–3.02	0.358
PF vs. TPF	2.60	1.30–5.20	0.007
GP vs. TPF	1.26	0.49–3.25	0.638
Others vs. TPF	1.82	0.76–4.40	0.182
**PROGRESSION-FREE SURVIVAL**			
**Radiotherapy**			
Moderate vs. radical RT	1.22	0.84–1.76	0.303
Palliative vs. radical RT	1.57	1.10–2.24	0.013
**Chemotherapy**			
TP vs. TPF	1.55	1.00–2.39	0.048
PF vs. TPF	1.78	1.15–2.74	0.009
GP vs. TPF	1.54	0.93–2.56	0.094
Others vs. TPF	1.86	1.10–3.17	0.022
***IN-SITU*** **BONE RELAPSE-FREE SURVIVAL**			
**Radiotherapy**			
Moderate vs. radical RT	1.80	0.76–4.31	0.184
Palliative vs. radical RT	3.46	1.57–7.66	0.002
**Chemotherapy**			
TP vs. TPF	3.21	1.05–9.86	0.042
PF vs. TPF	5.39	1.84–15.78	0.002
GP vs. TPF	2.71	0.76–9.62	0.123
Others vs. TPF	2.78	0.75–10.36	0.128

*HR, hazard ratio; CI, confidence interval; RT, radiotherapy; TP, paclitaxel plus cisplatin; PF, cisplatin plus 5-fluorouracil; GP, gemcitabine plus cisplatin; TPF, paclitaxel plus cisplatin and 5-fluorouracil*.

*The Cox proportional hazards model was used to perform the analysis*.

**Figure 3:**
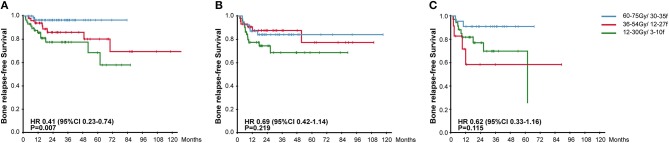
Kaplan-Meier Curves for Bone Relapse Free Survival among groups receiving different radiotherapy plans in patients with osteogenic lesions **(A)**, patients with osteolytic lesions **(B)**, and patients with soft tissue involvement **(C)**.

To further explore the effectiveness of radiotherapy plans on different types of BM, we separated patients according to the imaging features of BM destruction (osteogenic or osteolytic) and the absence/presence of soft tissue involvement. For patients with osteogenic BM, radical radiation plan significantly reduced the occurrence of *in-situ* relapse (96.6 vs. 83.3 vs. 76.4%, HR, 0.41, 95% CI, 0.23–0.74, *P* = 0.007). But in the meantime, radical radiotherapy failed to bring BRFS benefits to patients with osteolytic lesions or with soft tissue involvement (Figure 3).

### Systemic Therapy

Most of patients (91.7%) received platinum-based chemotherapy. The number and proportion of patients receiving TP, PF, GP, and TPF were 78 (25.4%), 75 (24.4%), 44 (14.3%), and 68 (22.1%), respectively. EGFR inhibitors such as cetuximab and nimotuzumab were administered to 59 (19.7%) patients, immune checkpoint inhibitors including CTLA-4, PD-1, and PD-L1 antibodies were given to 24 (8.0%) patients and anti-angiogenic agents to five (1.7%) patients. Furthermore, zoledronic acid was administered to 193 (64.3%) patients.

**Figure 4:**
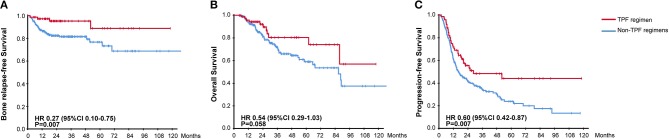
Kaplan-Meier Curves for **(A)** Bone Relapse Free Survival, **(B)** Overall Survival, **(C)** Progression Free Survival, between groups receiving or not receiving TPF chemotherapy regimen.

The efficacy of different chemotherapy regimens was compared and no significant difference apropos BRFS, OS, and PFS was found among TP, PF, and GP regimens. However, distinct BRFS (94.1 vs. 81.0%, HR, 0.27, 95% CI, 0.10–0.75, *P* = 0.007) and PFS (50.0 vs. 33.6%, HR, 0.60, 95% CI, 0.42–0.87, *P* = 0.007) benefits were achieved with the administration of TPF regimen compared to other chemotherapy plans (Figure 4). An improved OS on the boundary of significance was also observed (83.8 vs. 72.8%, HR, 0.54, 95% CI, 0.29–1.03, *P* = 0.058). As it was found that TPF was mostly administered to newly-diagnosed patients (Chi-square test, *P* < 0.001), we further explored the efficacy of TPF in newly-diagnosed cohort, and patients using the TPF regimen tended to exhibit better OS, PFS in contrast to other chemotherapy regimens (Supplementary Figure 1). The addition of targeted agents or immunotherapy helped to prolong the OS (88.6 vs. 69.8%, HR, 0.42, 95% CI, 0.22–0.82, *P* = 0.009), but didn't show superiority in terms of PFS and BRFS (Supplementary Figure 2). On the other hand, the application of zoledronic failed to bring any survival benefit to neither osteogenic nor osteolytic BM (data not shown).

**Figure 5:**
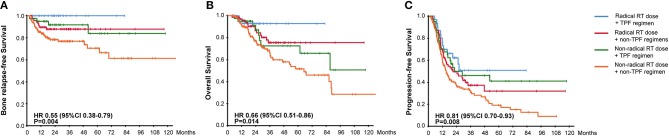
Kaplan-Meier Curves for **(A)** Bone Relapse Free Survival, **(B)** Overall Survival, **(C)** Progression Free Survival, among groups with different chemoradiotherapy.

To evaluate the combined effectiveness of chemoradiotherapy, we divided patients into 4 groups based on the aboved results. As the survival curves showed in Figure 5, the combination of radical radiotherapy and TPF regimen gave rise to the significantly improved BRFS, OS, and PFS. Meanwhile, the survival results were similar between patients who either received radical radiated dose or TPF regimen, followed by those who received neither of them.

### Univariable and Multivariable Analysis

Univariable analysis was performed first to screen out potential prognostic factors for BRFS and OS, and the multivariable analysis was made thereafter (Table 3). Compared with pretreatment EBV DNA level, whether EBV DNA could drop down to zero after RT offered more prognostic value, and stayed significant in the multivariable analysis for both BRFS and OS. It also indicated that the pre-treatment level of ALP served as an independent risk factor in the multivariable model for OS (HR, 3.50, 95% CI, 1.58–7.77, *P* = 0.002). With regard to the treatment strategy, the administration of TPF regimen failed to be a prognostic variant, but radiotherapy to BM with radical dose was proved as an independent protective factor for both BRFS (HR, 0.60, 95% CI, 0.36–0.98, *P* = 0.039) and OS (HR, 0.59, 95% CI, 0.35–0.98, *P* = 0.039).

**Table 3:** Univariable and multivariable analysis of prognostic factors for bone relapse-free survival and overall survival.

**Variables**	**Univariable**		**Multivariable**	
	**HR (95% CI)**	***P*-value**	**HR (95% CI)**	***P*-value**
**Bone relapse-free survival**				
Newly-diagnosed BM	0.37 (0.19–0.69)	0.002	0.45 (0.20–1.02)	0.054
Number of BM lesions	3.15 (1.77–5.62)	<0.001	2.07 (0.97–4.40)	0.059
Feature of BM destruction	1.47 (0.83–2.59)	0.183	§	
Concomitant metastasis in other organs	1.98 (1.10–3.56)	0.023	0.98 (0.46–2.09)	0.953
Pre-RT ALP level (U/L)	1.69 (0.79–3.64)	0.179	§	
Pre-RT LDH level (U/L)	1.54 (0.74–3.20)	0.273	§	
Pre-RT EBV DNA level (copies/ml)	1.31 (0.67–2.57)	0.434	§	
Post-RT EBV DNA level (copies/ml)	3.98 (1.97–8.01)	<0.001	2.39 (1.15–4.97)	0.020
Dose description of RT	0.53 (0.37–0.76)	0.001	0.60 (0.36–0.98)	0.039
Administration of TPF regimen	0.27 (0.10–0.75)	0.012	0.41 (0.12–1.36)	0.144
**Overall Survival**				
Newly-diagnosed BM	0.79 (0.50–1.26)	0.324	§	
Number of BM lesions	1.53 (0.96–2.44)	0.073	0.90 (0.39–2.09)	0.800
Feature of BM destruction	1.44 (0.91–2.28)	0.115	§	
Concomitant metastasis in other organs	1.55 (0.93–2.59)	0.091	1.53 (0.62–3.77)	0.352
Pre-RT ALP level (U/L)	1.87 (1.02–3.43)	0.044	3.50 (1.58–7.77)	0.002
Pre-RT LDH level (U/L)	1.00 (1.00–1.01)	0.045	1.21 (0.49–3.02)	0.679
Pre-RT EBV DNA level (copies/ml)	1.65 (0.89–3.07)	0.114	§	
Post-RT EBV DNA level (copies/ml)	2.84 (1.37–5.88)	0.005	2.30 (1.06–4.98)	0.035
Dose description of RT	0.63 (0.46–0.84)	0.002	0.59 (0.35–0.98)	0.039
Administration of TPF regimen	0.54 (0.29–1.03)	0.062	0.66 (0.24–1.84)	0.428

*Categorical variables were newly-diagnosed BM (yes vs. no); number of BM lesions (≤3 vs. >3); feature of BM destruction (osteolytic vs. osteogenic); concomitant metastasis in other organs (yes vs. no); pre-RT ALP level (≤110 vs. >110 U/L); pre-RT LDH level (≤250 vs. >250 U/L); pre-RT EBV DNA level (≤4,000 vs. >4,000 copies/ml); post-RT EBV DNA level (0 vs. >0 copies/ml); dose description of RT (60–75 Gy/30–35f vs. 36–54 Gy/12–27f vs. 12–30 Gy/3–10f); administration of TPF regimen (yes vs. no)*.

*HR, hazard ratio; CI, confidence interval; BM, bone metastasis; RT, radiotherapy; ALP, alkaline phosphatase; LDH, lactic dehydrogenase; EBV, Epstein-barr virus; TPF, paclitaxel plus cisplatin and 5-fluorouracil*.

§ *Variables were dropped out of the model*.

*P-values were calculated with the 2-sided Wald test in the Cox proportional hazard model*.

## Discussion

As good results have been achieved in the treatment of locoregionally advanced nasopharyngeal carcinoma, more attention should be paid to the management of metastatic NPC. The present study is the first one to explore the combinatorial treatment strategy for bone metastatic patients, and found that the intensity of dose prescription to the BM was strongly correlated to the BRFS, OS, and PFS. Meanwhile, the survival results in terms of BRFS, OS and PFS favored the administration of TPF regimen. Patients benefited most from the combination of radical radiation dose and TPF chemotherapy. Furthermore, radical radiation dose to the BM was proved to be an independent protective factor.

The appropriate dose prescription and fractionation to the BM has been discussed in many cancers. Although multiple studies demonstrated the equivalent effect of short fractionation schedules (including single fraction) on pain palliation, the situation might be different in treating NPC patients. As our results showed that 170 (56.7%) patients had asymptomatic BM, relieving the pain was not the major concern for giving radiotherapy. Besides, many patients presented with oligometastasis, and longer survival durations were often observed among them, thus the short fractionation schedules, which sometimes required the need for retreatment (18), were less adequate. Our results suggested the use of intensive dose prescription, which was consistent with the results in hepatocellular cancer (HCC), renal and prostate cancers (19–22). Kim et al. (20) selected HCC patients who were followed up for at least 1 year and a positive dose-response relationship was observed. In Koga et al.'s study (21), the intensive local therapy was beneficial not only for patients with solitary BM but also for multiple BMs. In our study, apart from reducing the chance of *in-situ* recurrence, the radical dose prescription significantly improved the OS and PFS. In summary, patients' predicted life expectancy and treatment goals should be considered when giving local radiotherapy. For patients with a chance of long-term survival, radical radiotherapy to the BM was preferred.

With respect to the optimum chemotherapy regimens for metastatic NPC, it had been inconclusive and the PF regimen was the most empirically-used regimen for over decades (23). Zhang et al. (24) carried out the first phase III trial to compare the efficacy of GP with PF regimen, and the results indicated that the GP regimen significantly prolonged the PFS in recurrent or metastatic NPC. However, conclusions in relative retrospective studies were slightly different. Jiang et al. (25) made comparisons among five cisplatin-based chemotherapy in metastatic NPC, and found significantly higher response rates were associated with the administration of GP and TPF regimens. Nonetheless, no difference was observed in OS and PFS, which might result from the less cycles of GP and TPF given to patients. In a meta-analysis which included 27 studies (26), triplet regimen demonstrated best short-term efficacy while TP regimen was associated with the highest 1 year OS. In the present study, TPF regimen was found superior to other regimens in terms of PFS and BRFS. However, a larger proportion of initially diagnosed patients receiving TPF regimen should be noticed. In multivariable analysis, the administration of TPF failed to be an independent protective factor. In that case, further prospective studies are still warranted to compare the effectiveness among chemotherapy regimens, especially between GP and TPF regimens. What's more, the administration of targeted therapy or immunotherapy shouldn't be neglected. Although the PFS rates were comparable between patients with or without targeted or immunotherapy, patients achieved significantly longer OS with the help of targeted or immunotherapy, which indicated the potential of them to prolong the survival after disease progression.

The study had several limitations. Primarily, because of the retrospective nature, selection bias may exist as clinicians were more inclined to give aggressive combinatorial treatment to patients with less metastatic burdens and satisfactory performance status. Secondly, given the scarcity of bone metastatic patients receiving local radiotherapy, we had to include patients with metastasis to other organs to guarantee the sample size for analysis, which might be a confounding variable while calculating PFS and OS. Furthermore, as the data was the representative of treating experience from single institute where the short-course hypofractionated (including single fraction) radiotherapy plans were seldomly used, the true efficacy of them in NPC patients remained unknown. Therefore, future studies which focus on the multi-modality treatment for bone metastatic NPC patients are in urgent need.

In conclusion, this study showed that the intensity of radiation dose to the BM was strongly associated with the BRFS, OS, and PFS, and it remained independently protective in multivariable analysis. The administration of TPF regimen might help to improve OS, PFS and BRFS compared with other platinum-based regimens, and patients benefited most from the combination of radical local radiation and TPF chemotherapy.

## Data Availability Statement

The datasets generated for this study are available on request to the corresponding author.

## Ethics Statement

This study was approved by the Sun Yat-sen University Cancer Center Clinical Research Ethics Committee.

## Author Contributions

H-QM and L-QT: study concepts. X-YL, J-JY, and G-DJ: study design. X-SS, S-LL, and S-SG: data acquisition. L-TL: quality control of data and algorithms. X-YL, X-SS, and G-DJ: data analysis and interpretation. X-SS and X-YL: statistical analysis. X-YL, RS, and D-HL: manuscript preparation. X-SS, S-LL, and H-YM: manuscript editing. Q-YC and LG: manuscript review.

### Conflict of Interest

The authors declare that the research was conducted in the absence of any commercial or financial relationships that could be construed as a potential conflict of interest. The reviewer XL declared a shared affiliation, with no collaboration, with the authors to the handling editor at time of review.

## Supplementary Material

Supplementary Figure 1 Kaplan-Meier Curves for **(A)** Bone Relapse Free Survival, **(B)** Overall Survival, **(C)** Progression Free Survival, between groups receiving or not receiving TPF chemotherapy regimen in newly-diagnosed patients.

Supplementary Figure 2 Kaplan-Meier Curves for **(A)** Bone Relapse Free Survival, **(B)** Overall Survival, **(C)** Progression Free Survival, between groups receiving or not receiving targeted therapy or immunotherapy.

Supplementary Table 1 Radiotherapy dose fractionation schedule for each case scenario.
