# Supplementary figures and images for: Intensive Local Radiotherapy Is Associated With Better Local Control and Prolonged Survival in Bone-Metastatic Nasopharyngeal Carcinoma Patients
Source: Front Oncol. 2020 Mar 20;10:378. doi: 10.3389/fonc.2020.00378 (PMC7100272; doi:10.3389/fonc.2020.00378)

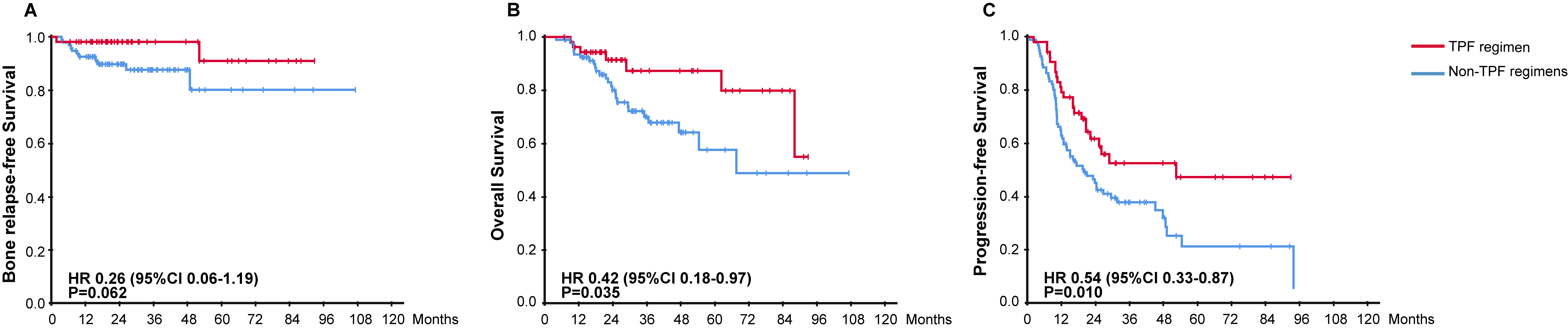

Supplement: Supplementary Figure 1 — Kaplan-Meier Curves for (A) Bone Relapse Free Survival, (B) Overall Survival, (C) Progression Free Survival, between groups receiving or not receiving TPF chemotherapy regimen in newly-diagnosed patients. [file Image_1.TIF]

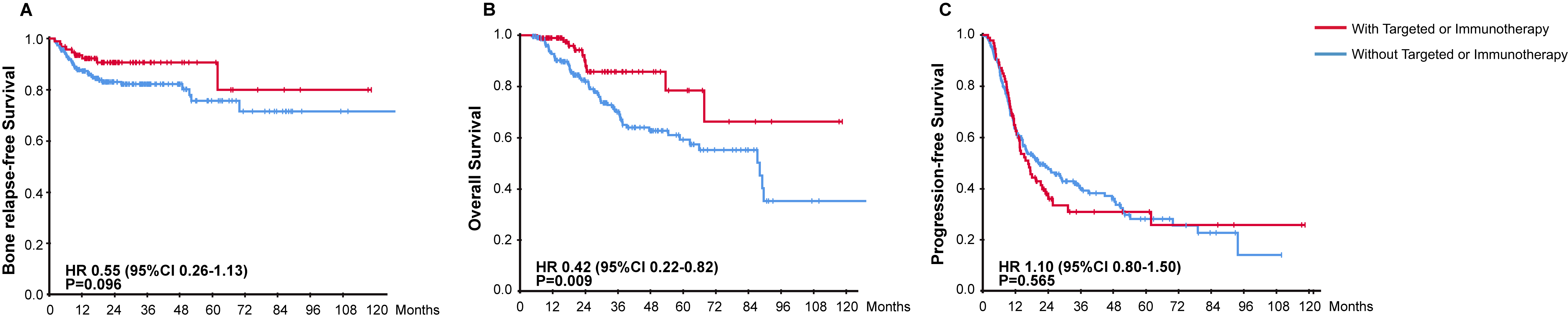

Supplement: Supplementary Figure 2 — Kaplan-Meier Curves for (A) Bone Relapse Free Survival, (B) Overall Survival, (C) Progression Free Survival, between groups receiving or not receiving targeted therapy or immunotherapy. [file Image_2.TIF]
