# Supplementary material for: Intensive Local Radiotherapy Is Associated With Better Local Control and Prolonged Survival in Bone-Metastatic Nasopharyngeal Carcinoma Patients
Source: Front Oncol. 2020 Mar 20;10:378. doi: 10.3389/fonc.2020.00378 (PMC7100272; doi:10.3389/fonc.2020.00378)
Supplement: Supplementary Table 1 — Radiotherapy dose fractionation schedule for each case scenario. [file Table_1.doc]

**Supplementary Table 1. Radiotherapy dose fractionation schedule for each case scenario**

| **Case** | **Accumulative dose (Gy)** | **Fractionation** | **Fractionated dose (Gy)** |
| --- | --- | --- | --- |
| 1 | 3.6 | 12 | 0.3 |
| 2 | 6 | 12 | 0.5 |
| 3 | 8 | 1 | 8.0 |
| 4 | 8 | 1 | 8.0 |
| 5 | 20 | 10 | 2.0 |
| 6 | 24 | 12 | 2.0 |
| 7 | 28 | 4 | 7.0 |
| 8 | 36 | 6 | 6.0 |
| 9 | 36 | 3 | 12.0 |
| 10 | 48 | 8 | 6.0 |
| 11 | 48 | 6 | 8.0 |
| 12 | 60 | 54 | 1.1 |
